# Variations of Surveillance Practice for Patients with Bone Sarcoma: A Survey of Australian Sarcoma Clinicians

**DOI:** 10.1155/2017/1837475

**Published:** 2017-02-28

**Authors:** Jeremy Lewin, Kate Thompson, Susie Bae, Jayesh Desai, Robyn Strong, Denise Caruso, Deborah Howell, Alan Herschtal, Michael Sullivan, Lisa Orme

**Affiliations:** ^1^Victorian Adolescent & Young Adult Cancer Service, Peter MacCallum Cancer Centre, Melbourne, VIC, Australia; ^2^Adolescent and Young Adult Oncology, Princess Margaret Cancer Centre, Toronto, ON, Canada; ^3^Australasian Sarcoma Study Group, Melbourne, VIC, Australia; ^4^Australian and New Zealand Children's Hematology/Oncology Group, Melbourne, VIC, Australia; ^5^Centre for Biostatistics and Clinical Trials, Peter MacCallum Cancer Centre, Melbourne, VIC, Australia; ^6^Children's Cancer Centre, Royal Children's Hospital, Melbourne, VIC, Australia; ^7^Department of Paediatrics, University of Melbourne, Melbourne, VIC, Australia

## Abstract

*Introduction*. After treatment, bone sarcoma patients carry a high chance of relapse and late effects from multimodal therapy. We hypothesize that significant variation in surveillance practice exists between pediatric medical oncology (PO) and nonpediatric medical oncology (NP) sarcoma disciplines.* Methods*. Australian sarcoma clinicians were approached to do a web based survey that assessed radiologic surveillance (RS) strategies, late toxicity assessment, and posttreatment psychosocial interventions*. Results*. In total, 51 clinicians responded. No differences were identified in local disease RS. In metastatic disease response assessment, 100% of POs (23/23) and 93% of NPs (24/26) conducted CT chest. However, this was more likely to occur for NPs in the context of a CT chest/abdomen/pelvis (NP: 10/26; PO: 1/23; *p* = 0.006). POs were more likely to use CXR for RS (*p* = 0.006). POs showed more prescriptive intensity in assessment of heart function (*p* = 0.001), hearing (*p* < 0.001), and fertility (*p* = 0.02). POs were more likely to deliver written information for health maintenance/treatment summary (*p* = 0.04). The majority of respondents described enquiring about psychosocial aspects of health (*n* = 33/37, 89%), but a routine formal psychosocial screen was only used by 23% (*n* = 6/26).* Conclusion*. There is high variability in bone sarcoma surveillance between PO and NP clinicians. Efforts to harmonize approaches would allow early and late effects recognition/intervention and facilitate improved patient care/transition and research.

## 1. Background

Sarcomas of the bone are rare primary malignant tumors arising from mesenchymal or neuroectodermal tissue and account for less than 1% of all malignancies [1]. The most common subtypes are osteosarcoma (OS) and Ewing's sarcoma family tumors (ESFT) with an average of 3000 new cases being diagnosed in the United States [2] and 200 cases being diagnosed in Australia each year [3]. Prior to the era of multimodality therapy, bone sarcomas were associated with an 80–90% risk of metastasis with resection alone [4]. However, advances in treatment have led to 5-year overall survival approximating 60–70% [5]. With improving survival, follow-up procedures for patients with bone sarcoma are becoming increasingly important, especially as primary treatment carries significant risks of long-term physical and psychological sequelae.

The aim of surveillance is multifold, including the potential to detect treatable recurrences, manage chronic toxicities, and screen for late effects. However, these benefits need to be tempered with healthcare expenditure concerns [6], anxiety associated with surveillance procedures, and cumulative radiation exposure from surveillance imaging [7]. Systematic, uniform, and sustainable targeted disease surveillance (DS) and long-term follow-up (LTFU) strategies that take these factors into account would allow evaluation and evidence generation regarding best practice in posttreatment follow-up. However, differences in established guidelines (which often do not translate into uniform practice) will potentially lead to significant variation in surveillance approaches. For example, guidelines written by the Children's Oncology Group (COG) [8], National Comprehensive Cancer Network (NCCN) [9], and European Society for Medical Oncology (ESMO) [10], which are all based on consensus opinion, vary slightly with regard to the optimal timing for DS, with pediatric protocols showing more concern for cumulative radiation exposure (Table 1). Thus it is likely that posttreatment surveillance schedules vary in regard to timing of clinical reviews, investigation choice, frequency, and duration, both between and within adult and pediatric sectors.

Previous US and UK based clinician surveys have demonstrated significant variation in follow-up procedures for patients with primarily soft tissue sarcoma [11–13]. This survey of Australian clinicians was undertaken to evaluate variations in bone sarcoma surveillance between exclusively pediatric based medical oncologists and nonexclusively pediatric based specialties (NP) and assist in informing the development of Australian guidelines for DS and LTFU strategies in bone sarcomas.

## 2. Materials and Methods

### 2.1. Recruitment of Respondents

As there was no definitive list of Australian clinicians involved in the surveillance of sarcoma patients, an overinclusive population of clinicians was identified via the internal databases of the Australasian Sarcoma Study Group (ASSG) and the Australian and New Zealand Children's Hematology Oncology Group (ANZCHOG). In addition, a manual search was conducted of clinicians involved in the major national sarcoma units. Participants were invited to participate via a web based survey (SurveyMonkey, Palo Alto, USA). Consent was assumed based on return response. The survey was open for a 4-month period between June and September 2015.

### 2.2. Survey Development

The cross-sectional survey was designed to assess a variety of domains associated with both disease and late effects surveillance of bone sarcoma patients. The complete survey is shown in Supplemental Appendix  1 (see Supplementary Material available online at https://doi.org/10.1155/2017/1837475). The 27 questions were distributed as follows: baseline demographics (e.g., type of practice, age demographic of patients, and case load); approach to end of treatment radiological assessment; approach to disease surveillance after primary treatment; approach to screening for late effects (e.g., blood work, ototoxicity, cardiotoxicity, and fertility); and practice for psychosocial support. Responses were designed based on available international surveillance protocols. In addition, respondents were given the opportunity to add comments in free text boxes to expand on specific details related to their particular practice. Before distribution, the questionnaire was piloted by a small group of clinicians. Reminder emails were sent at 2 time points to maximize the response rate.

### 2.3. Statistical Analysis

The data collected from SurveyMonkey was exported to the R statistical programming language for analysis [14]. Descriptive statistics were used to summarize characteristics of the respondents. Differences between PO and NPs were tested using Fisher's exact test for categorically valued policies and Wilcoxon's test for ordinal valued policies. Given that not all questions were mandatory, sample size varied according to the particular question. Thus responses have been displayed with the numerator (*n*) and denominator (*N*) (largest possible number of available responses). The denominator is reported for each section once, unless it changes. Statistical significance was set at a *p* value < 0.05 and all *p* values were 2-sided.

## 3. Results

### 3.1. Participants

The survey was sent to Australian clinician memberships of the ASSG (*n* = 228) and ANZCHOG (*n* = 129) database, of which 12 emails were undeliverable. In total, 51 clinicians responded (response rate = 15%). Demographics of the respondents are shown in Table 2. Of the 51 who responded, 23 (45%) were pediatric medical oncologists, 11 (22%) were adult medical oncologists, 9 (18%) were radiation oncologists, and 8 (16%) were surgical oncologists/orthopedic surgeons. The majority of respondents worked primarily in a public general hospital (*n* = 35, 69%). The number of respondents who saw pediatric, adolescent/young adult (AYA, defined as 15–25), and adult patients as part of their practice was 67%, 63%, and 47%, respectively. All surgeons worked in the AYA age group whereas 82% of medical oncologists, 56% of radiation oncologists, and 43% of pediatric oncologists worked in this age group. The majority of clinicians saw fewer than or equal to 10 new cases of bone sarcoma per year (69%) with higher case load (defined as >10 new cases per year) seen by orthopedic surgeons (6/7, 86%) and medical oncologists (6/11, 55%) compared to other disciplines (*p* = 0.009).

## 4. Disease Surveillance

### 4.1. Assessing Radiologic Response to Treatment

Preferences regarding end of treatment disease restaging investigations for localized limb bone sarcoma divided by discipline are shown in Table 3. With regard to assessment at the primary site, there were no differences identified in the use of CT, MRI, FDG-PET, and bone scan for assessing local disease. However, POs were more likely than NPs to use X-ray imaging alone or as part of pulmonary DS (*p* < 0.001). Regarding metastatic site response assessment, 100% of POs (*n* = 23, *N* = 23) and 93% of NPs (*n* = 24, *N* = 26) conducted CT chest; however, 10/26 NPs compared to 1/23 POs chose this as part of a CT chest/abdomen/pelvis study (*p* = 0.006). POs were more likely than NPs to use CXR in addition to CT chest (*p* = 0.02) and were more likely to include bone marrow aspirate (*p* = 0.006) (presumed with reference to Ewing sarcoma patients with bone marrow involvement at diagnosis; data not collected). There were no differences identified in the use of FDG-PET for assessing metastatic disease at the end of treatment (*p* = 0.55).

### 4.2. Radiologic Surveillance (RS) after Treatment

The relationship between posttreatment RS and discipline is shown in Table 4. There were no differences identified in the use of X-ray, MRI, CT, or FDG-PET for assessment of local recurrence when using either radiotherapy or surgery as the treatment modality. Several participants described that the type of surgical intervention was relevant to the disease surveillance strategy (e.g., in the setting of an amputation or the presence of a prosthetic implant (less likely to do MRI)). Additionally, some respondents commented that patients with cutaneous and superficial ESFT could be monitored clinically without primary site imaging. Regarding RS for metastatic disease, POs were more likely than NPs to use CXR for pulmonary surveillance (*p* = 0.006). Some respondents described alternating CXR and CT chest. There were no significant differences identified in the use of FDG-PET scans for metastatic disease, but typically this modality was used as a secondary investigation (i.e., to further investigate suspicious findings on CT). In addition, cumulative radiation exposure factored into the decision process for the RS modality being utilized (*n* = 31, *N* = 42; 74%) (e.g., CXR replacing CTs; the presence of* TP53* mutation; risk stratification with radiation minimization in low risk patients).

### 4.3. Frequency of Review

Overall, clinicians undertook 3-month follow-up (median) in years 1 and 2 and 6-month follow-up in years 3, 4, and 5, with no differences identified between disciplines. Differences between osteosarcoma and ES were identified in the free text analysis with clinicians described closer monitoring and longer lag time for relapse in ES patients. Others additionally described protocol related differences such as those mandated by COG.

### 4.4. Treatment Summary

When comparing POs to NPs, written information was more likely to be given to patients for health maintenance (47% versus 11%, *p* = 0.037). POs were also more likely to refer to a late effects clinic for complex patients (94% versus 44%, *p* = 0.03) (Table 5).

### 4.5. Duration of Surveillance

POs were more likely than NPs to follow up bone sarcoma patients for shorter time periods (5 years' duration: PO 69%, NP 26%; 10 years' duration: PO 13%, NP 43%; no endpoint: PO 19%, NP 22%; *p* = 0.04).

## 5. Screening for Late Effects

### 5.1. Blood Work

**Figure 1:**
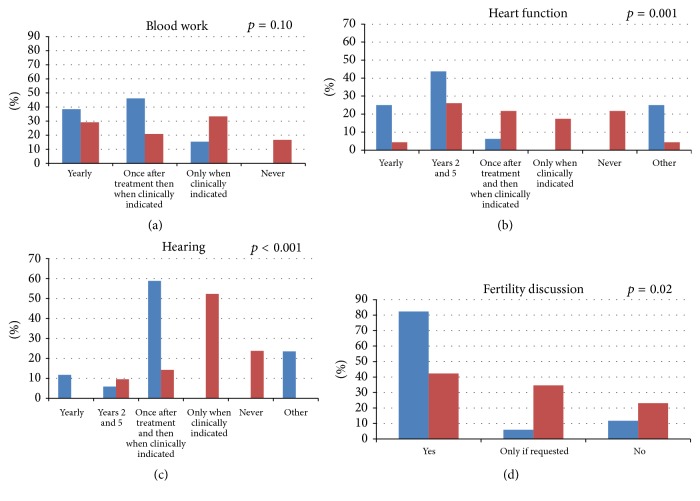
Timing of blood work (a), heart function assessment (b), hearing assessment (c), and fertility discussion (d). *p* values represent comparison between PO (blue) and NP disciplines (red).

No differences were identified in the utilization of blood work with 38% of POs (*n* = 5, *N* = 13) and 29% of NPs (*n* = 7, *N* = 24) drawing blood yearly and 46% of POs (*n* = 6, *N* = 13) and 21% of NPs (*n* = 5, *N* = 24) drawing blood once after treatment and then when clinically indicated (*p* = NS) (Figure 1(a)). 17% of NPs (*n* = 4, *N* = 24) did not undertake screening blood work. The role of screening blood work conducted by radiation oncologists was described in the context of checking biochemistry if the kidney and/or liver were within the radiotherapy field. Although not specifically asked, some clinicians described undertaking screening urinary cytology for those receiving alkylating agents.

### 5.2. Cardiotoxicity, Ototoxicity, and Fertility

POs were more likely to have more prescriptive intensity in assessing heart function with multiple heart function assessment occurring in 94% of POs compared with 32% in NPs (*p* = 0.001) (Figure 1(b)). In addition to standard evaluations, clinicians described other factors that influenced the timing of heart scans such as cumulative doxorubicin dose, previous chest irradiation, according to established guidelines (e.g., COG), and in the setting of pregnancy. Similarly, the assessment of ototoxicity was more intensive by POs with 77% assessing hearing function at least once after treatment compared to 24% of NPs (*p* < 0.001) (Figure 1(c)). Two respondents described that ototoxicity screening may occur through external programs and thus surveillance procedures occur outside of sarcoma clinics. Routine fertility discussions were more likely to be conducted by POs (82%) than NPs (42%) (*p* = 0.02) (Figure 1(d)).

### 5.3. Psychosocial Support

Although the overwhelming majority of respondents describe routinely enquiring about psychosocial aspects of health (*n* = 33, *N* = 37, 89%), a routine formal psychosocial screen was only used in 23% (*n* = 6, *N* = 26). The majority of respondents had the support of allied health (e.g., social worker, physiotherapy, and psychology) and nursing services to assess psychosocial support needs for patients off treatment and manage/refer accordingly (*n* = 24, *N* = 31, 77%).

## 6. Discussion

Posttreatment surveillance in patients with bone sarcoma aims to detect asymptomatic recurrences and to manage the chronic toxicities associated with primary treatment. However, the rarity of bone sarcoma and limited research in this area have led to significant practice variation amongst Australian clinicians involved in the follow-up of these patients.

Follow-up for bone sarcoma patients serves a number of purposes. The primary aim is to identify asymptomatic recurrences early enough to initiate treatment that will lead to improved outcomes. However, currently it is unclear whether RS is associated with improved outcomes [15–18], and opinion is divided, with only 67% of musculoskeletal tumor surgeons believing that early relapse detection led to improved survival [13]. It is currently unknown whether that belief holds true with nonsurgical clinicians. Nevertheless, in this survey there was wide consensus on the utility of RS and the timing of follow-up was consistent with intervals described by international guidelines [8–10]. Of note, POs were more likely than NPs to follow-up bone sarcoma patients for shorter time periods. However, this may be accounted for by transition of care to adult centers in AYA patients and more frequent referral to dedicated late effects service.

A major concern of intensive DS is the cumulative radiation exposure and corresponding concerns of secondary malignancy [7, 19, 20]. As a result, a general trend has been seen with the use of low dose protocol in children and improved scanning techniques that may result in reduced radiation exposure [8, 21]. Reflecting this, over 70% of respondents altered the timing or modality of RS on the basis of patient concerns, hereditary disposition syndromes, or based on risk stratification approaches. Not unexpectedly, POs were more likely to adopt an RS schedule cognisant of radiation exposure. They were thus more likely to alternate CT chest with CXR in RS for pulmonary metastasis (*p* = 0.02) and less likely to conduct full body CTs in end of treatment response assessments (*p* = 0.003). Given that pulmonary metastasis is the usual site of distant metastatic spread, there is arguably no role for routine whole body CTs. In addition, Australian PO's practice departs from established COG guidelines in that DS had variable inclusion of functional imaging which is recommended by COG. Of note, the only published trial comparing differing RS approaches in bone and soft tissue sarcoma did not demonstrate any differences in outcome with reduced intensity, either in the timing (3 versus 6 months) or in the modality (CXR versus CT) [18]. However, as this study included all extremity sarcomas (including low grade lesions with lower metastatic potential), it is unknown whether exchanging modalities and reducing radiation exposure are acceptable in high grade bone sarcoma where relapse rates are considerably higher. Although not specifically addressed in this survey (but reflected in COG guidelines [8]), the case for routine surveillance using CT chest in ESFT is less compelling. This is because relapses in ESFT are usually multifocal, compared to OS, where surgical salvage alone is likely to be appropriate, making early detection advantageous.

Regarding screening of local disease, previous reports demonstrated a high rate of patient detection of local relapse. In the TOSS trial [18], 90% of local recurrences were identified on the basis of symptoms. This is in keeping with other similar reports showing low rates of asymptomatic radiological detection [16, 17]. Although most clinicians in this survey described conducting RS of the local site without significant variation in modality, the timing/frequency of such undertaking were not explored. The question of whether these tests were conducted for assessing disease relapse or for other reasons such as prosthetic alignment was also not addressed.

In addition to DS, identifying and managing late effects of primary treatment are an important aspect of sarcoma surveillance care. Most of the literature assessing LTFU has stemmed from pediatric survivorship research. This is due to the many decades survivors of pediatric cancers live after intensive treatments and given that these treatments are delivered at an age where critical growth and maturation of organ systems are taking place [22–24]. It was consequently not unexpected to discover variations in practice identified with screening for late effects (with more prescriptive intensity in screening for ototoxicity, cardiotoxicity, and fertility discussions by POs). However, these variations in practice are important, as AYAs may be managed in adult based surveillance programs. They may therefore be treated according to lines of a more elderly cancer population, with limited thought given beyond cure. The potential for late effects is considerable with the Childhood Cancer Survivor Study showing that 62% of survivors of pediatric cancer had at least one chronic condition and 28% had a severe or life-threatening condition at a median of 17.5 years after diagnosis [22]. Thus a unifying late effect surveillance approach, aligned across pediatric, and adult institutions would allow early recognition and intervention thus minimizing the frequency of severe complications [25]. Variation in late effects surveillance may also exist because POs are supported by pediatric based international guidelines, which present exposure and risk-based guidelines for management of late effects [26]. Of note, adult based surveillance sarcoma guidelines such as those from NCCN [9] and ESMO [10] do not expand on specific surveillance in the timing or mechanisms for LTFU screening. This is an important underserved area as late effects are of considerable concern to those in survivorship (e.g., infertility risks [27]) and empowering patients with unified LTFU plans is important for self-care management. Risk stratification and arming low risk patients with treatment summaries/suggested late effects surveillance that can be enacted by local doctors may assist in overcoming practice variation and allow late effects resources to focus on high risk patients. However, even if adult based guidelines were developed targeting late effects screening, it is likely that practice variations would continue. This is due to the lack of uniformity in resource allocation, with subsequent effects on AYA service provision and availability of late effects clinics.

Although this survey identified that 89% of the respondents routinely enquired about psychosocial aspects of health, few undertook systemic psychosocial screening. Given the peak incidence of sarcoma in the AYA years [28], developing systems, such as screening tools, that proactively identify those at increased risk of psychological distress or those who need intervention/assistance is imperative. There are many aspects of a new cancer diagnosis that may be particularly confronting for AYAs. These include premature confrontation with mortality, disruption in the capacity to feel “normal,” increased dependency on parents, social life interference, and fertility concerns. Developmentally appropriate targeted support for this age group is therefore required [29, 30]. However, assessing the specific overall quality of life (QOL) specifically in bone sarcoma patients is difficult with significant heterogeneity in patient populations (age, tumor type) and instrumentation used in established QOL literature [31]. Mechanisms for routine systematic screening of AYAs are currently in development [32]. However, with the increasing costs associated with implementing AYA programs and screening tools, it is unclear as to when to implement them, how they should be validated, and how to evaluate their cost-effectiveness.

We acknowledge several limitations in this survey. Firstly, although the number of participants is similar to previous sarcoma surveys [11–13], the corresponding overall response rate of 15% is low. This is driven by the large denominator of nonpracticing clinicians/general oncologists on the ASSG and ANZCHOG database who are likely not involved in sarcoma surveillance. Although we aimed to be overinclusive, the low response rate does raise the potential for nonresponse bias. Counteracting this weakness is that this survey focused exclusively on bone sarcoma clinicians with a focus on looking at variations in practice between POs and NP specialties. In addition, it is unclear as to whether this information is generalizable to clinicians at large, as current practices in Australia may not reflect global clinician practices. Similarly, survey amalgamation of RS questions for EFST/OS and site of disease (e.g., pelvic versus limb) does not allow for variations of practice in subtypes of bone sarcoma. Furthermore, clinicians were not enquired as to whether patients' insurance status had an effect on surveillance preferences. Lastly, given the small numbers of individual subspecialties, comparisons between individual groups and analysis of variation within groups could not be undertaken.

## 7. Conclusion

In conclusion, despite a relatively small and specialized field, this survey of Australian clinicians has identified significant variation in practice with regard to DS and LTFU for patients with bone sarcoma. A uniform holistic approach is important for patients treated with bone sarcoma as coherent pathways empower patients and families irrespective of age of diagnosis, place of care, or identified clinician. Lack of uniformity puts AYA patients undergoing transition at risk of disengaging. Therefore, confident transition plans are required between pediatric and adult centers. DS requires a sensible, radiation cognizant schedule adopted in line with current international guidelines and appropriate to local health system contexts. The impetus for its development should be generated from pediatric and adult multidisciplinary collaborative efforts and the development of such a standardized guideline should be mindful of the paucity of evidence base supporting practice. Efforts to harmonize DS and LTFU approach between pediatric and adult centers, factoring in available evidence, treatment protocols, cost, and radiation exposure, would allow early late effects recognition and intervention and facilitate improved patient care/transition, data collection, and research.

## Supplementary Material

Supplemental Appendix 1. Cross-sectional online survey of Australian clinicians regarding disease and late effects surveillance of bone sarcoma patients.

## Figures and Tables

**Table 1:** Differences in RS protocols between COG, NCCN, and ESMO.

	COG [8]		NCCN [9]		ESMO [10]	
	OS	ES	OS	ES	OS and ES	For ES only
Frequency	(i) Primary site: every 3 months × 8 (yr 0–2) and then every 6 mo × 6 (yr 3–5) and then every 12 months × 5 (yr 6–10) (ii) Chest: CT chest every 3 mo × 8 (yr 0–2) and then every 6 mo × 2 (yr 3) and then every 12 months × 2 (yr 4-5). CXR, every 12 months × 5 (starting after last scheduled CT, yr 6–10)	(i) Primary site: every 3 months × 8 (yr 0–2) and then every 6 mo × 6 (yr 3–5) and then every 12 months × 5 (yr 6–10) (ii) Chest: CXR every 3 mo × 8 (yr 0–2) and then every 6 mo × 6 (yr 3–5) and then every 12 months × 5 (yr 6–10)	(i) Every 3 mo (yr 0–2) (ii) Every 4 mo (yr 3) (iii) Every 6 months (yr 4-5) (iv) Every year (>yr 5)	Every 2-3 months for 2 years and then increasing intervals up to 5 years and then annually indefinitely	(i) Every 2-3 mo (yr 0–2) (ii) Every 2–4 mo (yr 3-4) (iii) Every 6 mo (yr 5–10) (iv) Every 6–12 mo (>yr 10)	

Modality	(i) Primary site: AP and lateral X-ray (ii) Chest: CT chest and CXR (iii) Bone scan if symptoms or abnormal imaging (iv) FDG-PET if symptoms or abnormal imaging (v) MRI or CT primary if symptoms or abnormal imaging	(i) Primary site: AP and lateral X-ray (ii) Chest: AP and lateral (iii) Bone scan if symptoms or abnormal imaging (iv) FDG PET if symptoms or abnormal imaging (v) Chest CT if abnormal CXR (vi) MRI or CT primary if symptoms or abnormal imaging	(i) Physical exam (ii) Laboratory studies (iii) Chest imaging (iv) Imaging of primary site (v) Consider FDG/PET or bone scan (vi) Functional assessment at every visit	(i) Physical exam (ii) Laboratory studies (iii) Cross-sectional imaging (MRI with or without CT) and X-ray of primary site (iv) Chest CT (v) Consider PET/CT or bone scan	(i) Physical exam (ii) Laboratory studies (iii) Functional assessment (iv) Imaging of primary site (v) CXR/CT chest	Bone scan can be added

**Table 2:** Baseline demographics.

		Number (%)
Discipline	Pediatric medical oncology	23 (45%)
	Adult medical oncology	11 (22%)
	Radiation	9 (18%)
	Orthopaedics	7 (14%)
	Other	1 (2%)

Age demographic	Exclusively pediatric^*∗*^	15 (29%)
	Exclusively adult^*∗*^	4 (8%)
	Exclusively AYA	2 (4%)
	Pediatric as part of practice	34 (67%)
	Adult as part of practice	24 (47%)
	AYA as part of practice	32 (63%)

Institutional location	Private practice	2 (4%)
	Private general hospital	2 (4%)
	Public general hospital	36 (71%)
	Oncology specific hospital	11 (22%)

Volume of new patients per year	0–10	35 (69%)
	11–20	12 (24%)
	20–30	1 (2%)
	30–50	2 (4%)
	>50	1 (2%)

^*∗*^Including Adolescent and Young Adult (AYA), defined as 15–25 years old.

**Table 3:** Approach to end of treatment radiological assessment.

	PO	Med Onc	Radiation	Ortho	*p* value^*∗∗*^	OR	OR 95% CI
Number of responses	23	10	9	7			
End of treatment response assessment							
Local disease							
X-ray	20 (87%)	3 (30%)	2 (22%)	2 (29%)	<0.001	14.78	[3.15, 99.95]
CT	5 (22%)	2 (20%)	1 (11%)	2 (29%)	1.00	0.97	[0.20, 4.57]
MRI	18 (78%)	7 (70%)	9 (100%)	6 (86%)	0.72	0.63	[0.11, 3.42]
FDG-PET	16 (70%)	5 (50%)	7 (78%)	3 (43%)	0.56	1.56	[0.42, 6.07]
Bone scan	6 (26%)	0 (0%)	0 (0%)	1 (14%)	0.12	4.28	[0.66, 48.30]
Metastatic disease							
CXR	7 (30%)	0 (0%)	0 (0%)	0 (0%)	0.02	10.87	[1.22, 530.27]
CT chest	23 (100%)	5 (50%)	5 (56%)	4 (57%)	<0.001	*∗*	[3.47, *∗*]
CT chest/abdomen/pelvis	1 (4%)	4 (40%)	3 (33%)	3 (43%)	0.006	0.07	[0.00, 0.56]
FDG-PET	15 (65%)	8 (80%)	7 (78%)	4 (57%)	0.55	0.66	[0.16, 2.62]
Whole body scintigraphy	5 (22%)	0 (0%)	0 (0%)	1 (14%)	0.23	3.39	[0.49, 39.36]
Whole body MRI	0 (0%)	0 (0%)	0 (0%)	0 (0%)	1.00	*∗*	[0.00, 45.75]
Bone marrow aspirate	6 (26%)	0 (0%)	0 (0%)	0 (0%)	0.006	*∗*	[1.60, *∗*]

^*∗*^Inestimable due to insufficient data.

^*∗∗*^ *p* value represents comparison between PO and NP.

**Table 4:** Approach to radiological surveillance.

	PO	Med Onc	Radiation	Ortho	*p* value^*∗∗*^	OR	OR 95% CI
Number of responses	17	10	9	7			
Radiologic surveillance after treatment							
Local disease (after surgery)							
X-ray	12 (71%)	4 (40%)	2 (22%)	5 (71%)	0.12	2.97	[0.70, 14.29]
CT	6 (35%)	3 (30%)	1 (11%)	1 (14%)	0.31	2.14	[0.43, 11.20]
MRI	13 (76%)	8 (80%)	8 (89%)	6 (86%)	0.41	0.45	[0.06, 3.14]
FDG-PET	7 (41%)	2 (20%)	2 (22%)	3 (43%)	0.51	1.77	[0.40, 8.00]
Local disease (after radiation)							
X-ray	5 (29%)	3 (30%)	1 (11%)	1 (14%)	0.48	1.73	[0.32, 9.28]
CT	4 (24%)	3 (30%)	1 (11%)	0 (0%)	0.69	1.67	[0.26, 10.68]
MRI	16 (94%)	8 (80%)	9 (100%)	5 (71%)	0.63	2.85	[0.25, 151.94]
FDG-PET	7 (41%)	1 (10%)	4 (44%)	3 (43%)	0.53	1.56	[0.36, 6.73]
Metastatic disease							
CXR	9 (53%)	2 (20%)	1 (12%)	0 (0%)	0.006	7.77	[1.47, 56.24]
CT chest	13 (76%)	7 (70%)	6 (75%)	2 (29%)	0.33	2.13	[0.47, 11.61]
Alternating CXR and CT	7 (41%)	2 (20%)	1 (12%)	3 (43%)	0.31	2.17	[0.48, 10.34]
CT C/A/P	0 (0%)	0 (0%)	1 (12%)	2 (29%)	0.26	*∗*	[*∗*, 3.52]
FDG-PET	6 (35%)	5 (50%)	4 (50%)	4 (57%)	0.35	0.51	[0.12, 2.10]
Bone scan	0 (0%)	0 (0%)	1 (12%)	1 (14%)	0.51	*∗*	[*∗*, 7.84]

^*∗*^Inestimable due to insufficient data.

^*∗∗*^ *p* value represents comparison between PO and NP.

**Table 5:** Late effects monitoring.

	PO	Med Onc	Radiation	Ortho	*p* value^*∗*^	OR	OR 95% CI
Number of responses	17	10	9	7			
Late effects clinic for complex patients	16 (94%)	4 (40%)	8 (89%)	4 (57%)	0.03	9.56	[1.12, 459.37]
Referral to GP/relevant subspecialists	11 (65%)	8 (80%)	8 (89%)	0 (0%)	1.00	1.14	[0.27, 5.04]
Give written information about health maintenance/lifestyle to patient	8 (47%)	1 (10%)	3 (33%)	0 (0%)	0.04	4.69	[0.97, 27.15]
Give verbal information about health maintenance/lifestyle	9 (53%)	6 (60%)	5 (56%)	1 (14%)	0.76	1.3	[0.33, 5.32]

^*∗*^ *p* value represents comparison between PO and NP.
