# Supplementary material for: Variations of Surveillance Practice for Patients with Bone Sarcoma: A Survey of Australian Sarcoma Clinicians
Source: Sarcoma. 2017 Feb 28;2017:1837475. doi: 10.1155/2017/1837475 (PMC5350324; doi:10.1155/2017/1837475)
Supplement: Supplementary file 1 — Supplemental Appendix 1. Cross-sectional online survey of Australian clinicians regarding disease and late effects surveillance of bone sarcoma patients. [file 1837475.f1.pdf]

Dear Clinician,

Guidelines including those created by the NCCN, ESMO and COG for disease surveillance and long term follow up of bone sarcomas are empirically based. Consequently it is likely that there is considerable variation in the frequency, duration and modality of follow-up in bone sarcomas. This survey seeks to explore current practices in disease surveillance and long term follow up among health care providers treating bone sarcomas across Australia and New Zealand. It should take approximately 10-15 minutes. The questionnaire answers are confidential and this project has been approved by the Peter MacCallum Cancer Centre's REB.

We request that you forward this on to other colleagues who also treat bone sarcomas. The survey is not restricted to individuals working in specialised institutes but also aims to reach a range of sarcoma providers across the oncology community. This will enable us to better understand the spectrum of approaches for disease surveillance and long term follow up.

Dr Lisa Orme

Dr Jeremy Lewin

OnTrac @ PeterMac

Baseline Demographics

*1. Which discipline are you qualified to practice in?

- ○ Paediatric Oncology
- ○ Medical Oncology
- ○ Radiation Oncology
- ○ Surgical Oncology/Orthopaedics

Other (please specify)

*2. In which age demographic do you practice? Tick all that apply

- ☐ Paediatric (0-18yo)
- ☐ Adult
- ☐ Adolescent and Young Adult (15-25yo)

3. In which sort of institution do you primarily work?

- ○ Private practice
- ○ Private General Hospital
- ○ Public General Hospital
- ○ Oncology Specific Hospital

Other (please specify)

Baseline Demographics

4. Approximately what volume of new bone sarcoma patients do you personally treat, per year?

- 0-10
- 11-20
- 21-30
- 30-50
- >50

Other (please specify)

5. As part of your practice do you routinely provide disease surveillance reviews once a patient has completed treatment?

- Yes
- No

6. If you conduct disease surveillance, which disciplines are involved other than yourself?

- ☐ surgical oncology/orthopedic
- ☐ medical oncology
- ☐ radiation oncology
- ☐ no other disciplines involved
- ☐ pediatric oncology
- ☐ N/A

Other (please specify)

7. If you DO NOT conduct disease surveillance, who is responsible for disease surveillance for your bone sarcoma patients?

- ☐ Surgical Oncology/Orthopedic
- ☐ Radiation Oncology
- ☐ Medical Oncology
- ☐ Pediatric Oncology
- ☐ N/A

Other (please specify)

Approach to end of treatment response

8. For patients with localized limb bone sarcoma, which imaging modality do you use for end of treatment restaging to assess response at the primary site? Select all that apply

- [ ] Xray
- [ ] CT
- [ ] MRI
- [ ] FDG-PET
- [ ] Bone scan

Other (please specify)

9. For patients with localized limb bone sarcoma, which imaging modality do you use for end of treatment restaging to assess the presence of metastatic disease? Select all that apply

- [ ] CT chest
- [ ] CT Chest/Abdo/Pelvis
- [ ] FDG-PET
- [ ] Whole body scintigraphy
- [ ] Whole body MRI
- [ ] CXR
- [ ] Bone Marrow Aspirate/biopsy

Other (please specify)

Approach to disease surveillance

10. Do you routinely conduct radiological surveillance in asymptomatic individuals who are under surveillance for bone sarcomas?

- ○ Yes
- ○ No, as organized by colleagues in another discipline
- ○ No, as do not believe in radiological surveillance in asymptomatic individuals
- ○ Sometimes (please explain in comment box)

Sometimes (please specify)

11. In your experience, what is the ideal frequency of clinical review for bone sarcoma patients in the first 5 years post treatment? Please respond with monthly intervals

First year

Second year

Third year

Fourth year

Fifth year

12. In your experience, is there a difference between Ewings sarcoma and Osteosarcoma for frequency of disease surveillance in the first 5 years post treatment?

- ○ No
- ○ Yes

If answered Yes, please comment on change in practice

13. At the end of active treatment, what is your routine practice for giving patients a treatment summary and surveillance schedule?

- ○ No routine practice
- ○ Routine verbal explanation
- ○ Routine written/electronic copy

Other (please specify)

Approach to disease surveillance

14. If you conduct radiological surveillance to screen for local recurrence after a surgical procedure as primary local therapy, what modality/modalities of imaging do you use?

- [ ] Xray
- [ ] MRI
- [ ] CT
- [ ] FDG-PET
- [ ] Don't conduct radiological surveillance

Please comment if differences exist between OS and ESFT and whether you risk stratify disease

15. If you conduct radiological surveillance to screen for local recurrence after definitive radiation as primary local therapy what modality of imaging do you use?

- [ ] Xray
- [ ] MRI
- [ ] CT
- [ ] FDG-PET
- [ ] Don't conduct radiological surveillance

Please comment if differences exist between OS and ESFT and whether you risk stratify disease

16. Does the ability to clinically examine the primary site of local disease have bearing on whether you conduct radiological surveillance for local recurrence?

- ○ Yes (please explain in comment box)
- ○ No
- ○ Sometimes (please explain in comment box)

please specify

17. In patients with previous localised bone sarcoma, which imaging modalities to you use to screen for metastatic disease? (tick all that apply)

- ☐ CXR
- ☐ CT chest
- ☐ Alternating CXR and CT
- ☐ Bone scan
- ☐ CT C/A/P
- ☐ Whole body MRI
- ☐ FDG-PET
- ☐ Don’t conduct radiological surveillance

Are there differences between osteosarcoma and Ewing's sarcoma in your scanning approach for metastatic disease? (please comment)

18. Does the radiation exposure associated with imaging alter the timing and/or modality of radiological surveillance you conduct?

- ○ Yes
- ○ No
- ○ Sometimes (eg patients concern, suspected hereditary predisposition)

Comment (please specify)

Approach to disease surveillance

19. Do you routinely do blood work as part of surveillance for late effects in bone sarcoma patient?

- Once post treatment and then when clinical indicated
- Never
- Yearly
- Only when clinically indicated
- At year 2 and 5

Other (please specify)

20. After a patient has received anthracyclines, do you assess heart function?

- Once post treatment and then when clinically indicated
- Never
- Yearly
- Only when clinically indicated
- At years 2 and 5

Other (please specify)

21. After a patient has received ototoxic agents, how often do you assess hearing function?

- Once post treatment and then when clinical indicated
- Never
- Yearly
- Only when clinically indicated
- At year 2 and 5

Other (please specify)

22. What is your practice with regard to provision of OR a referral for psychosocial support for off therapy patients undergoing disease surveillance?

	Yes	No
I have no routine practice but if a patient brings up an issue during a review appointment I will discuss and refer to appropriate individuals	☐	☐
I routinely enquire about psychosocial aspects of health and refer accordingly	☐	☐
Allied health or nursing staff are available to assess psychosocial support needs for patients off treatment and manage/refer accordingly	☐	☐
There is a routine formal psychosocial screen for all patients off treatment	☐	☐

Other (please specify)

23. When psychosocial support is required for an outpatient in disease follow up, who do you usually refer to?

- ○ No routine practice
- ○ Hospital allied health team (eg Social worker, Physiotherapy, Psychology)
- ○ Correspond with General Practitioner to facilitate community based services
- ○ Recommend patient initiate community based services

Comments or please specify if other

24. Do you routinely conduct a fertility assessment/fertility discussion for those in disease surveillance who have finished primary therapy?

- ○ Yes
- ○ No
- ○ Only if requested

25. If fertility discussion occurs during a disease surveillance consultation who makes the referral to a fertility specialist?

- nurse coordinator or nurse practitioner
- you, the clinician
- patient needs to organize
- local doctor

Other (please specify)

Approach to disease surveillance

26. What do you believe is the duration of disease surveillance that should occur?

- ○ 5 years
- ○ 10 years
- ○ 15 years
- ○ 20 years
- ○ No defined endpoint

Other (please specify) and comment regarding differences between osteosarcoma and ewings sarcoma

27. At the end of disease surveillance, what is your approach to ongoing late effects monitoring? select all that apply

- ☐ Late effects clinic for complex patients
- ☐ GP/relevant subspecialists
- ☐ Give written information about health maintenance/lifestyle to patient
- ☐ Give verbal information about health maintenance/lifestyle
- ☐ No specific approach
